# Prevalence and Phylogenetic Analysis of Microsporidium *Enterocytozoon bieneusi* in Diarrheal Patients

**DOI:** 10.3390/pathogens10020128

**Published:** 2021-01-27

**Authors:** Manman Zang, Jinjin Li, Chun Tang, Songtao Ding, Wei Huang, Qizhong Qin, Handeng Liu

**Affiliations:** 1 Laboratory of Tissue and Cell Biology, Experimental Teaching and Management Center, Chongqing Medical University, Chongqing 400016, China; 2016220184@stu.cqmu.edu.cn (M.Z.); 2016210050@stu.cqmu.edu.cn (J.L.); 2016220130@stu.cqmu.edu.cn (C.T.); weide@vip.163.com (S.D.); qqizhong@cqmu.edu.cn (Q.Q.); 2 College of Pediatrics, Chongqing Medical University, Chongqing 400016, China; 3 College of Clinical, Chongqing Medical University, Chongqing 400016, China; 4 Chongqing Center for Disease Control and Prevention, Chongqing 400042, China; hw827@163.com

**Keywords:** *Enterocytozoon bieneusi*, genotype, microscopic examination, patient, phylogeny

## Abstract

*Enterocytozoon bieneusi* can cause severe diarrhea in children and adults. However, in China, there are scant studies on *E. bieneusi* in diarrheal children and adults, with the exception of prevalence and genotyping data in a small number of cities including Hubei, Shanghai, and Heilongjiang. In this study, 196 fecal samples (*n* = 132 in Chongqing, *n* = 44 in Shandong, *n* = 20 in Hubei) were collected, including 91 from children and 105 from adults. Through microscopic examination, 19 positive samples (11 from children and 8 from adults) were detected. Using PCR examination, the internal transcriptional spacer (ITS) region was utilized by nested PCR to detect and characterize *E. bieneusi*. Twenty positive samples were detected, including 14 from children (≤11 years of age) and 6 from adults. According to the sequence analysis of ITS data, one known zoonotic (D) and seven novel (CQH5-11) genotypes were identified. This is the first molecular epidemiological study of *E. bieneusi* in diarrheal patients in different regions of China. Therefore, this study can provide useful information for the molecular epidemiology and control of *E. bieneusi* infection in humans in the future.

## 1. Introduction

Microsporidia has more than 200 genera and 1500 species, among which 17 species of 9 genera can infect humans. *Enterocytozoon bieneusi* is the most common type diagnosed in humans, among which 90% of microsporidia infections are caused by *Enterocytozoon bieneusi* [1,2,3]. In 1985, *E. bieneusi* was first described as an opportunistic intestinal pathogen associated with HIV, and its morphology was characterized by electron microscopy. In 1996, morphologically identical spores were found in pig feces, which were subsequently discovered in intestinal tissues and fecal samples of other mammals [4,5]. To date, *E. bieneusi* infection has been detected in 236 animal species [6].

*Enterocytozoon bieneusi* is a common etiological agent of diarrhea in humans and animals around the globe. It can infect many vertebrates, including mammals, birds, amphibians, and reptiles and causes high morbidity and mortality in immunocompromised people. There is a wide range of sources of infection in the environment, especially in wild, domestic, and farm animals, and surface water. At the same time, *E. bieneusi* spores have been widely found in drinking water, soil, and livestock and wildlife. Furthermore, mature spores have thick walls, which make them resistant to chlorine at concentrations used to disinfect drinking water. Zoonotic transmission is the main source of infection and can occur either directly by contact with infected animals or humans with inadequate sanitation, or via ingesting water or food contaminated with the pathogens indirectly.

So far, most published studies of *E. bieneusi* have involved adults or children with diarrhea [7,8,9], but the correlation between the infection rate and age remains uncertain. Therefore, we conducted this research to get more information about the prevalence of *E. bieneusi* in diarrheal children and adult patients.

## 2. Results

**Figure 1:**
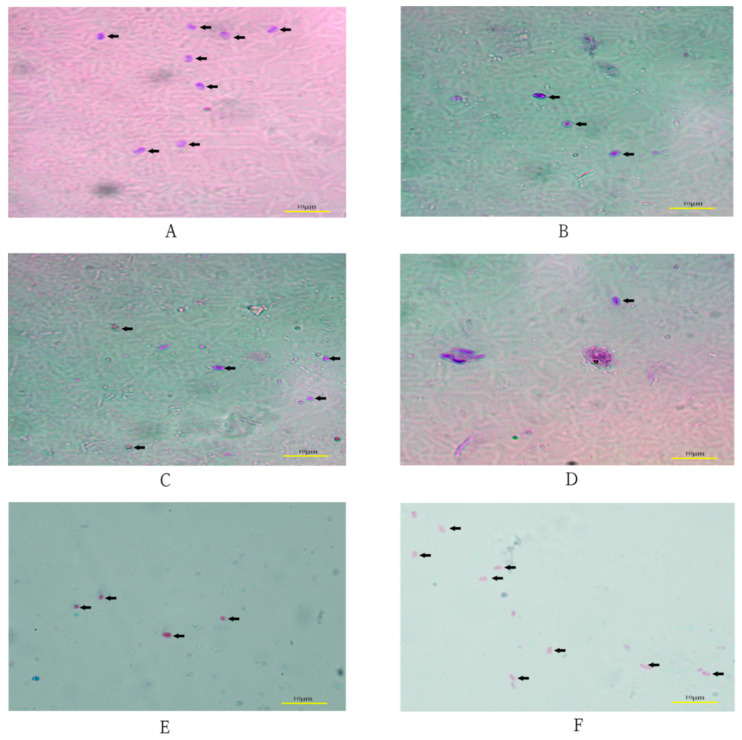
*Enterocytozoon bieneusi* detected from diarrheal stool specimens by microscopic examination. (**A**–**F**) were six different samples. The arrows indicate spores of *E. bieneusi*. *Scale bar* = 10 μm.

In this study, 196 fresh stool samples (91 from children (≤11 years old) and 105 from adults) were collected. Using microscopic examination and PCR amplification methods, some positive samples were detected among these stool samples. *Enterocytozoon bieneusi* stained with modified trichrome blue appears bright pink. A posterior vacuole and central diagonal pink stripe are visible within several *E. bieneusi* spores in Figure 1. A total of 19 positive samples (11 from children and 8 from adults) were detected by microscopic examination (Table 1), and 20 positive samples (14 from children and 6 from adults) were detected by PCR examination.

The prevalence of *E. bieneusi* was 9.70% (by microscope) and 10.20% (by PCR) (Table 1). Univariate analysis showed that although the prevalence of *E. bieneusi* was higher in children (12.09%, 11/91; 15.38%, 14/91) than adults (7.62%, 8/105; 5.71%, 6/105), and the prevalences in samples from Qingzhou (11.36%, 5/44), in Shandong, and Chongqing (9.09%, 12/132; 10.61%, 14/132) were slightly higher than that of Suizhou, in Hubei, there was no statistical difference in the infection rate of *E. bieneusi* among people of different ages (χ^2^ = 2.186, *p* > 0.05) and regions (χ^2^ = 0.614, *p* > 0.05).

**Figure 2:**
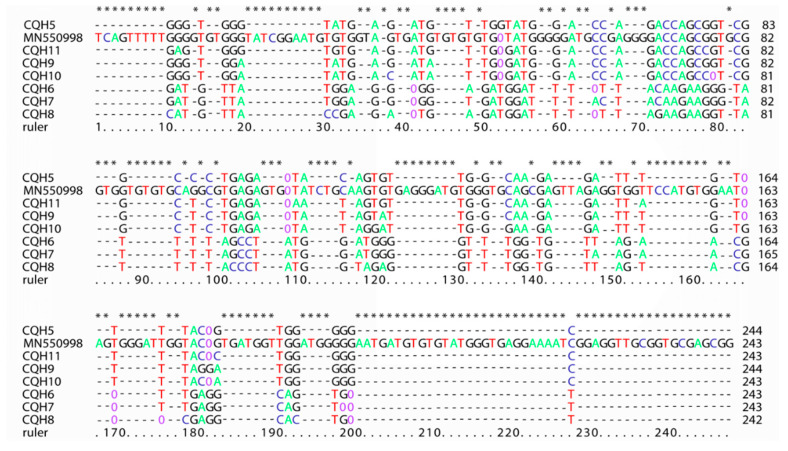
Sequence differences of internal transcriptional spacer (ITS) sequences obtained in this study. “-” indicates an identical nucleotide to MN550998, “0” indicates a missing nucleotide.

Among all these positive samples, 10 samples were sequenced successfully. In the end, only eight internal transcriptional spacer (ITS) (~243 bp) sequences (two sequences were identified according to other sequenced results) were obtained. According to the naming convention for genotypes of *E. bieneusi* [10], analysis of these ITS sequences revealed eight genotypes, including one known genotype (D) and seven new genotypes (called CQH5-11). The accession number for genotype D is MN550998. Sequence differences of ITS sequence and relatives among these seven new *E. bieneusi* genotypes in this research are shown in Figure 2. Among all the eight ITS sequences obtained in this study, there are 88 polymorphic positions including insertion/deletion, transition, and transversion with MN550998 as the reference sequence.

**Figure 3:**
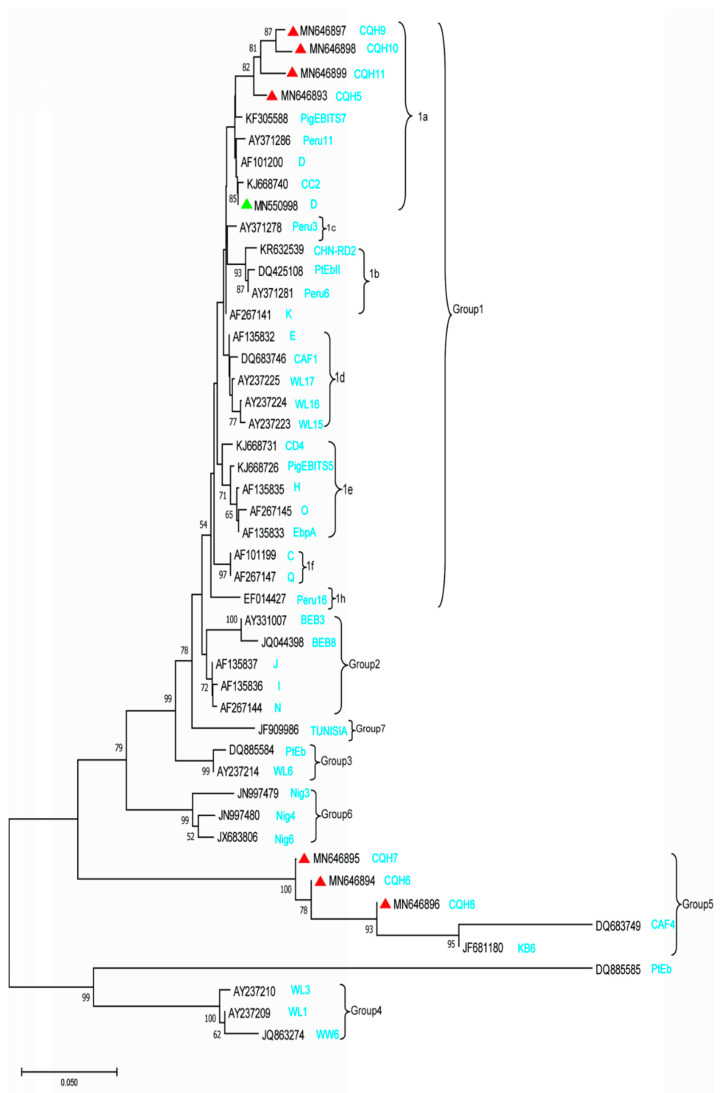
The phylogenetic tree was based on the neighbor-joining analysis of ITS sequences. The phylogenetic relationships of *E. bieneusi* genotypes determined here and other known genotypes previously deposited in GenBank were inferred by a neighbor-joining analysis of ITS sequences based on genetic distance by the Kimura 2-parameter model. The numbers on the branches are percent bootstrapping values from 1000 replicates. Each sequence is identified by its accession number, host origin, and genotype designation. The novel genotypes identified in this study are indicated with red triangles, and the known genotypes are indicated with a green triangle.

In order to obtain the information on the ITS sequences in this study and the reference ITS sequences published in previous studies, a phylogenetic tree was constructed. Phylogenetic analysis revealed that the eight *E. bieneusi* genotypes detected in this research formed two genetic clusters, with genotypes D, CQH5, CQH9, CQH10, and CQH11 clustered into Group 1, while genotypes CQH6, CQH7, and CQH8 clustered into Group 5 (Figure 3).

## 3. Discussion

As of October 2020, in China, there are a total of nine articles on the study of *E. bieneusi* infection in humans. The prevalence of *E. bieneusi* in the Chinese population changed from 0.2% to 22.5% [11]. The first report was Changchun City in Northeast China in 2011, which reported the infection in diarrheal children (9/40, 22.5%) and pigs (10/61, 16.4%) [9]. The infection of *E. bieneusi* was 4.2% (29/683) and 5.7% (39/683) in HIV-negative patients and HIV-positive patients in Henan province [12], respectively. Additionally, the following rates of infection were observed in other studies on other regions in China: 1.18% (27/2284) in children in Zhengzhou [13], 5.9% (36/609) in Xinjiang [14], 2.5% (3/121) in Daqing [15], 0.2% (1/500) in children in Wuhan [16], 13.49% (34/252) in adults and children of a diarrhea clinic in Shanghai [17], 11.83% (11/93) in children in Chongqing [18], and 11.58% (33/285) in HIV-positive patients in Guangxi [19]. According to statistics, the prevalence of *E. bieneusi* is 6.4% in southern China, and 5.5% in northern China [11], which is consistent with our results. The overall prevalence is 6.4%, 8.1%, and 3.6% for diarrhea patients, HIV patients, and healthy individuals in China, respectively. In previous studies, it was determined that immunocompromised patients, such as cancer patients or organ transplant legatees, as well as children, the elderly, and travelers were more likely to be infected with *E. bieneusi* [20,21]. In a cross-sectional study conducted throughout Thailand, the infection rate of *E. bieneusi* was notably higher in children aged 3–15 years (3.0%) than in participants aged >15 years (0.4%) (*p* = 0.0258) [22]. However, in our study, there was no statistical difference in the infection rate of *E. bieneusi* between diarrheal children (≤11 years) and adults, indicating that diarrheal children (≤11 years) and adults were equally likely to be infected with *E. bieneusi*. We conjecture that the incidence of diarrhea in children is higher than adults, so the prevalence of *E. bieneusi* between diarrheal children (≤11 years) and adults presents no significant difference.

ITS is the only known polymorphic marker in *E. bieneusi,* which shows very high variability compared to other microsporidia [23]. Therefore, currently, sequencing based on the internal transcribed spacer (ITS) region of the rRNA gene is the standard method for genotyping *E. bieneusi* [24]. More than 470 genotypes have been identified by this method in humans, mammals, birds, and water, including 59 human-specific and 31 zoonotic genotypes. In China, there are 41 genotypes (including genotype D, I, J, CHN1, CHN2, CHN3, CHN4, EbpC, type IV, Peru8, Peru11, PigEBITS7, Henan-I, Henan-II, Henan-III, Henan-IV, Henan-V, CS-4, NEC1, NEC2, NEC3, NEC4, NEC5, GX25, GX456, GX458, CQH1, CQH2, CQH3, CQH4, A, CHN6, EbpA, KB-1, NIA1, CXJH1, CXJH2, CXJH3, J, BEB6, and CM8) that have been identified in humans [9,12,13,14,15,16,17,18,19]. Phylogenetic analysis of the ITS sequence revealed the presence of at least 11 different genetic clusters, named as Groups 1 to 11 [25]. Group 1, the largest group, contains 314 genotypes and is further divided into nine subgroups named 1a to 1i. In previous studies, the first group is considered to have zoonotic potential because many genotypes are closely related to a wide range of hosts, and 94% of the isolates belonged to this group, including humans, suggesting the possible transmission between humans and animals. Group 2, the second largest group, was divided into three subgroups designated as 2a to 2c [10,26,27,28,29]. Group 3 consists of three sequences of musk rats (WL4 to WL6) and one cat sequence, while Group 4 consists of three different sequences of raccoons (WL1 to WL3) [30]. Another group that infects human is Group 5, including genotype CAF4, which was found in HIV-positive patients in Gabon and HIV-negative patients in Cameroon. CAF4 was the first genotype that was found among all genotypes isolated from humans that did not belong to Group 1 [31]. Group 6 includes genotypes found in municipal wastewater and raccoons; Group 7 includes genotypes found in HIV-positive patients in Nigeria [32]. In this study, seven new genotypes were firstly discovered in humans. CQH5, CQH9, CQH10, and CQH11 were distributed in Group 1 subtype 1a, indicating that these genotypes have a high probability of zoonotic transmission and public health importance. Despite the knowledge about molecular phylogeny being extensive, the full range of host diversity, including reservoirs and zoonotic transmission, remains unresolved. Genotype D and EbpC were identified as prevalent genotypes. In this study, a total of eight genotypes were found, seven of which were newly discovered genotypes, and were firstly discovered in humans. The remaining one was genotype D, which was the most common type found in domestic HIV patients (3.86%, 11/285) [19]. However, due to the lack of domestic research on human samples, genotype D is more common in animals, rabbit (0.94%, 4/426) [33], fox (23.04%, 44/191), raccoon dogs (8.64%, 14/162) [34], water deer (35.4%, 17/48) [35], donkey (4.17%, 2/48) [36], and squirrel (12.50%, 18/144) [37]. The seven new genotypes identified provide extra insights into the genotypic diversity in *E. bieneusi.* In addition, there are a wide variety of polymorphisms among these eight ITS sequences (Figure 2). Because only the polymorphisms at the ITS region can be used in designating new genotypes [10], the ITS diversity in this study may supply more information to *E. bieneusi* research.

In other countries, multiple studies have used MLST (multilocus sequence typing) tools to further explore the genetic variations in ITS genotypes in human and have already found 67, 25, 29, and 37 haplotypes at MS1, MS3, MS4, and MS7, respectively [6,38]. In one study, two different MLGs (multilocus genotypes) were identified as the same ITS genotype (BEB6) by MLST, suggesting that the use of a single genetic location is inadequate to determine whether two isolates are similar enough to be considered identical. However, up to now, there has been no report on MLST analysis of *E. bieneusi* in humans in China. Therefore, MLST should be used to systematically reveal the population structure and genetic polymorphism of *E. bieneusi* in future.

As a zoonotic pathogen, *E. bieneusi* causes human and animal diseases (both in livestock and companion animals). Due to the extensive range of hosts, multiple genotypes can coexist in humans and animals, and it can be widely detected in urban sewage, which is a huge hidden danger to public health. Currently, it is considered to be an opportunistic pathogen, but several outbreaks have occurred in immunocompetent humans and wild animals. At the same time, the number of susceptible people, such as AIDS patients, children, and the elderly, have been increasing year by year, and it will cause a certain burden on society in the future. At present, no effective vaccines and drugs have been developed for *E. bieneusi*, suggesting that more measures must be taken to minimize the threat of this pathogen on public health.

## 4. Materials and Methods

### 4.1. Ethics Statement

The protocol of this study was reviewed and approved by the Research Ethics Committee and the Animal Ethical Committee of Chongqing Medical University. All fecal samples in this research were collected under the permission of the patients or the parents of diarrheic children.

### 4.2. Sample Collection

Between February, 2017 and November, 2019, 196 fresh stool samples were donated to us by the First Affiliated Hospital of Chongqing Medical University and the Children’s Hospital of Chongqing Medical University (*n* = 68), the Chongqing Center for Disease Control and Prevention (*n* = 64), the People’s Hospital of Qingzhou in Shandong (*n* = 44), and the Hongshan Hospital of Suizhou in Hubei (*n* = 20) from diarrhea patients (different ages and genders). The specimens were collected from patients clinically diagnosed with diarrhea, and with fecal excretion heavier than 200 mg and no less than three events of diarrhea per day. Samples were all collected and placed in a 15-mL centrifuge tube and frozen at −20 °C.

### 4.3. Microscopic Examination

Microscopic examination of microsporidia in feces was performed by the modified trichrome staining method [39]. The staining solution in this study was prepared by dissolving 6 g of chromotrope 2R (BBI Life Sciences, Shanghai, China) with 0.5 g of aniline blue (Solarbio Life Sciences, Beijing, China) and 0.7 g of phosphotungstic acid in 3 mL of glacial acetic acid. This solution stood at room temperature for 30 min, after which 100 mL of distilled water was added and 1 M/L HCl was added to generate a pH 2.5 solution. Methanol-fixed smears were stained in this chromotrope 2R solution for 30 min at 37 °C and then rinsed for 10 s with acid alcohol (4.5 mL of acetic acid in 995.5 mL of 90% ethyl alcohol). The smears were then dehydrated with a 10-s rinse in 95% ethyl alcohol, two 5-min incubations in 95% ethyl alcohol, a 10-min incubation in 100% ethyl alcohol, and a 5-min incubation in xylene (or xylene substitute). Then these smears were examined under a microscope.

### 4.4. DNA Extraction and Separation

Before DNA extraction, the fecal specimens from humans were washed three times with distilled water in a centrifuge tube and concentrated by differential centrifugation. The pellet was retained for DNA extraction. QIAamp DNA Stool Mini Kit (QIAgen, Hilden, Germany) was used for genomic DNA extraction, which has been shown to be highly effective at removing PCR inhibitors and yield higher amounts of DNA [40,41]. The method we used was according to the manufacture’s recommendations after a slight improvement [42]. The extracted DNA was stored in a refrigerator at −20 °C until analyzed by PCR.

### 4.5. PCR Amplification

The internal transcriptional spacer (ITS) gene of *E. bieneusi* were identified by using nested PCR. The primers used in this study were as previously described [43]. Takara TaqTM (TaKaRa Bio Inc., Tokyo, Japan) was used as a premix that contains Taq DNA Polymerase, dNTP mixture, Taq buffer, and MgCl_2_. Each 50-μL reaction mixture contained 5 μL of 5 μM sense and antisense primers each, 1 uL of DNA template, 26.75 μL of nuclease-free water, and 12.25 μL of Taq mixture. All nested PCR products were detected by 1.2% agarose gel electrophoresis and visualized by UV-Bluing. All PCR amplifications included both a positive (human DNA sample) and negative control (distilled water) and were performed in duplicate. The PCR products of expected size were sequenced directly in both directions on an ABI 3730xl automated DNA sequencer (Invitrogen, Guangzhou, China). The original samples of the positive products have been retained.

### 4.6. Sequences Difference and Phylogenetic Analysis

Sequences most similar to those obtained here were identified by BLAST analysis on the NCBI GenBank database and aligned using the ClustalX 1.83 program. Comparing the ITS regions of all sequences obtained with the reference sequences downloaded from the GenBank database, the diversity of genotypes and the genetic relationship between new genotypes and known genotypes was illustrated. A phylogenetic tree was constructed using the neighbor-joining method [44] implemented in software MEGA X (https://www.megasoftware.net/) [45] to explain their genetic relationship, and the evolutionary distances were calculated by Kimura two-parameter model. The reliability of cluster formation was evaluated by the bootstrap method with 1000 replicates. Bootstrap values above 70% were shown in this study.

### 4.7. Statistical Analysis

Chi-square tests were used to assess the association between *E. bieneusi* test-positivity and factors such as age and location. Odds ratios (ORs) and 95% confidence intervals (95% CI) were used to measure the univariate associations. In this study, *p*-values of <0.05 were considered statistically significant. All statistical analyses were performed using SPSS Statistics 23.0 (International Business Machines Corporation, New York, NY, USA) (www.ibm.com/products/spss-statistics).

### 4.8. Nucleotide Sequence Accession Numbers

Eight *E. bieneusi* genotypes were obtained in this study, seven of which were newly discovered genotypes. The nucleotide sequence numbers of the ITS have been uploaded to the GenBank database, accession numbers MN646893–MN646899.

## Figures and Tables

**Table 1:** *Enterocytozoon bieneusi* detection of fecal samples by microscope and PCR methods.

Locations		Chongqing		Suizhou, Hubei	Qingzhou, Shandong	
		Adult	Children	Adult	Adult	Children
Number of samples		68	64	20	17	27
Positive ^a^ (%)	microscope	5 (7.35%)	7 (10.94%)	2 (10%)	1 (5.88%)	4 (14.81%)
	PCR	5 (7.35%)	9 (14.06%)	1 (5%)	0	5 (18.52%)

^a^ Not all of the positive samples were sequenced successfully.

## Data Availability

This data can be found here: [The National Center for Biotechnology Information https://www.ncbi.nlm.nih.gov/ GenBank Accession Number: MN646893–MN646899].

## References

[1] Santín M., Fayer R. (2011). Microsporidiosis: *Enterocytozoon bieneusi* in domesticated and wild animals. Res. Vet. Sci..

[2] Ramanan P., Pritt B.S. (2014). Extraintestinal microsporidiosis. J. Clin. Microbiol..

[3] Ghoyounchi R., Ahmadpour E., Spotin A., Mahami-Oskouei M., Rezamand A., Aminisani N., Ghojazadeh M., Berahmat R., Mikaeili-Galeh T. (2017). Microsporidiosis in Iran: A systematic review and meta-analysis. Asian Pac. J. Trop. Med..

[4] Mathis A., Weber R., Deplazes P. (2005). Reviews: July 2005, Volume 18, Issue 3 Author’s Correction: Zoonotic Potential of the Microsporidia. Clin. Microbiol. Rev..

[5] Kwak D., Seo M.G. (2020). Genetic analysis of zoonotic gastrointestinal protozoa and microsporidia in shelter cats in South Korea. Pathogens.

[6] Wang S.S., Wang R.J., Fan X.C., Liu T.L., Zhang L.X., Zhao G.H. (2018). Prevalence and genotypes of *Enterocytozoon bieneusi* in China. Acta Trop..

[7] Khanduja S., Ghoshal U., Agarwal V., Pant P., Ghoshal U.C. (2017). Identification and genotyping of *Enterocytozoon bieneusi* amonghuman immunodeficiency virus infected patients. J. Infect. Public Health.

[8] Desportes I., Le Charpentier Y., Galian A., Bernard F., Cochand-Priollet B., Lavergne A., Ravisse P., Modigliani R. (1985). Occurrence of a new microsporidan: *Enterocytozoon bieneusi* n. g., n. sp., in the Enterocytes of human patient with AIDS. J. Eukaryot. Microbiol..

[9] Zhang X., Wang Z., Su Y., Liang X., Sun X., Peng S., Lu H., Jiang N., Yin J., Xiang M. (2011). Identification and genotyping of *Enterocytozoon bieneusi* in China. J. Clin. Microbiol..

[10] Santín M., Fayer R. (2009). *Enterocytozoon bieneusi* genotype nomenclature based on the internal transcribed spacer sequence: A consensus. J. Eukaryot. Microbiol..

[11] Qiu L., Xia W., Li W., Ping J., Ding S., Liu H. (2019). The prevalence of microsporidia in China: A systematic review and meta-analysis. Sci. Rep..

[12] Wang L., Zhang H., Zhao X., Zhang L., Zhang G., Guo M., Liu L., Feng Y., Xiao L. (2013). Zoonotic cryptosporidium species and *Enterocytozoon bieneusi* genotypes in HIV-positive patients on antiretroviral therapy. J. Clin. Microbiol..

[13] Yu F., Li D., Chang Y., Wu Y., Guo Z., Jia L., Xu J., Li J., Qi M., Wang R. (2019). Molecular characterization of three intestinal protozoans in hospitalized children with different disease backgrounds in Zhengzhou, central China. Parasites Vectors.

[14] Qi M., Yu F., Zhao A., Zhang Y., Wei Z., Li D., Zhang L. (2020). Unusual dominant genotype NIA1 of *Enterocytozoon bieneusi* in children in Southern Xinjiang, China. PLoS Negl. Trop. Dis..

[15] Yang J., Song M., Wan Q., Li Y., Lu Y., Jiang Y., Tao W., Li W. (2014). *Enterocytozoon bieneusi* genotypes in children in northeast China and assessment of risk of zoonotic transmission. J. Clin. Microbiol..

[16] Wang T., Fan Y., Koehler A.V., Ma G., Li T., Hu M., Gasser R.B. (2017). First survey of *Cryptosporidium*, *Giardia* and *Enterocytozoon* in diarrhoeic children from Wuhan, China. Infect. Genet. Evol..

[17] Liu H., Shen Y., Yin J., Yuan Z., Jiang Y., Xu Y., Pan W., Hu Y., Cao J. (2014). Prevalence and genetic characterization of *Cryptosporidium*, *Enterocytozoon*, *Giardia* and *Cyclospora* in diarrheal outpatients in china. BMC Infect. Dis..

[18] Ding S., Huang W., Qin Q., Tang J., Liu H. (2018). Genotype Identification and Phylogenetic Analysis of *Enterocytozoon bieneusi* Isolates from Stool Samples of Diarrheic Children. J. Parasitol..

[19] Liu H., Jiang Z., Yuan Z., Yin J., Wang Z., Yu B., Zhou D., Shen Y., Cao J. (2017). Infection by and genotype characteristics of *Enterocytozoon bieneusi* in HIV/AIDS patients from Guangxi Zhuang autonomous region, China. BMC Infect. Dis..

[20] Didier E.S., Weiss L.M. (2011). Microsporidiosis: Not just in AIDS patients. Curr. Opin. Infect. Dis..

[21] Zhang W., Ren G., Zhao W., Yang Z., Shen Y., Sun Y., Liu A., Cao J. (2017). Genotyping of *Enterocytozoon bieneusi* and subtyping of *Blastocystis* in cancer patients: Relationship to diarrhea and assessment of zoonotic transmission. Front. Microbiol..

[22] Prasertbun R., Mori H., Sukthana Y., Popruk S., Kusolsuk T., Hagiwara K., Mahittikorn A. (2019). *Enterocytozoon bieneusi* and *Cryptosporidium*: A cross-sectional study conducted throughout Thailand. BMC Infect. Dis..

[23] Henriques-Gil N., Haro M., Izquierdo F., Fenoy S., del Aguila C. (2010). Phylogenetic approach to the variability of the microsporidian *Enterocytozoon bieneusi* and its implications for inter- and intrahost transmission. Appl. Environ. Microbiol..

[24] Palareti G., Legnani C., Cosmi B., Antonucci E., Erba N., Poli D., Testa S., Tosetto A. (2016). Comparison between different D-Dimer cutoff values to assess the individual risk of recurrent venous thromboembolism: Analysis of results obtained in the DULCIS study. Int. J. Lab. Hematol..

[25] Dengjel B.M., Zahler M., Hermanns W., Heinritzi K., Spillmann T., Thomschke A., Loscher T., Gothe R., Rinder H. (2001). Zoonotic Potential of *Enterocytozoon bieneusi*. J. Clin. Microbiol..

[26] Reetz J., Rinder H., Thomschke A., Manke H., Schwebs M., Bruderek A. (2002). First detection of the microsporidium *Enterocytozoon bieneusi* in non-mammalian hosts (chickens). Int. J. Parasitol..

[27] Sulaiman I.M., Bern C., Gilman R., Cama V., Kawai V., Vargas D., Ticona E., Vivar A., Xiao L. (2003). A Molecular Biologic Study of *Enterocytozoon bieneusi* in HIV-Infected Patients in Lima, Peru. J. Eukaryot. Microbiol..

[28] Sulaiman I.M., Fayer R., Yang C., Santin M., Matos O., Xiao L. (2004). Molecular characterization of *Enterocytozoon bieneusi* in cattle indicates that only some isolates have zoonotic potential. Parasitol. Res..

[29] Santín M., Trout J.M., Fayer R. (2005). *Enterocytozoon bieneusi* genotypes in dairy cattle in the eastern United States. Parasitol. Res..

[30] Sulaiman I.M., Fayer R., Lal A.A., Trout J.M., Schaefer F.W., Xiao L. (2003). Molecular characterization of microsporidia indicates that wild mammals harbor host-adapted *Enterocytozoon* spp. as well as human-pathogenic *Enterocytozoon bieneusi*. Appl. Environ. Microbiol..

[31] Yakoob J., Abbas Z., Beg M.A., Jafri W., Naz S., Khalid A., Khan R. (2012). Microsporidial infections due to *Encephalitozoon intestinalis* in non-HIV-infected patients with chronic diarrhoea. Epidemiol. Infect..

[32] Karim M.R., Dong H., Li T., Yu F., Li D., Zhang L., Li J., Wang R., Li S., Li X. (2015). Predomination and new genotypes of *Enterocytozoon bieneusi* in captive nonhuman primates in zoos in China: High genetic diversity and zoonotic significance. PLoS ONE.

[33] Zhang X.X., Jiang J., Cai Y.N., Wang C.F., Xu P., Yang G.L., Zhao Q. (2016). Molecular characterization of *Enterocytozoon bieneusi* in domestic rabbits (*Oryctolagus cuniculus*) in northeastern China. Korean J. Parasitol..

[34] Yang Y., Lin Y., Li Q., Zhang S., Wei T., Wan Q., Jiang Y., Li W. (2015). Widespread presence of human-pathogenic *Enterocytozoon bieneusi* genotype D in farmed foxes (*Vulpes vulpes*) and raccoon dogs (*Nyctereutes procyonoides*) in China: First identification and zoonotic concern. Parasitol. Res..

[35] Amer S., Kim S., Han J.I., Na K.J. (2019). Prevalence and genotypes of *Enterocytozoon bieneusi* in wildlife in Korea: A public health concern. Parasites Vectors.

[36] Yue D.M., Ma J.G., Li F.C., Hou J.L., Zheng W.B., Quan Z., Zhang X.X., Zhu X.Q. (2017). Occurrence of *Enterocytozoon bieneusi* in donkeys (*Equus asinus*) in China: A public health concern. Front. Microbiol..

[37] Deng L., Li W., Yu X., Gong C., Liu X., Zhong Z., Xie N., Lei S., Yu J., Fu H. (2016). Correction: First report of the human-pathogenic *Enterocytozoon bieneusi* from red-bellied tree squirrels (*Callosciurus erythraeus*) in Sichuan, China. PLoS ONE.

[38] Li W., Cama V., Feng Y., Gilman R.H., Bern C., Zhang X., Xiao L. (2012). Population genetic analysis of *Enterocytozoon bieneusi* in humans. Int. J. Parasitol..

[39] Didier E.S., Orenstein J.M., Aldras A., Bertucci D., Rogers L.B., Janney F.A. (1995). Comparison of three staining methods for detecting microsporidia in fluids. J. Clin. Microbiol..

[40] Hawash Y. (2014). DNA extraction from protozoan oocysts/cysts in feces for diagnostic PCR. Korean J. Parasitol..

[41] Mirsepasi H., Persson S., Struve C., Andersen L.O.B., Petersen A.M., Krogfelt K.A. (2014). Microbial diversity in fecal samples depends on DNA extraction method: EasyMag DNA extraction compared to QIAamp DNA stool mini kit extraction. BMC Res. Notes.

[42] Menu E., Mary C., Toga I., Raoult D., Ranque S., Bittar F. (2018). Evaluation of two DNA extraction methods for the PCR-based detection of eukaryotic enteric pathogens in fecal samples. BMC Res. Notes.

[43] Buckholt M.A., Lee J.H., Tzipori S. (2002). Prevalence of *Enterocytozoon bieneusi* in swine: An 18-month survey at a slaughterhouse in Massachusetts. Appl. Environ. Microbiol..

[44] Saito N., Nei M. (1987). The neighbor-joining method: A new method for reconstructing phylogenetic trees. Mol. Biol. Evol..

[45] Kumar S., Stecher G., Li M., Knyaz C., Tamura K. (2018). MEGA X: Molecular evolutionary genetics analysis across computing platforms. Mol. Biol. Evol..

